# CSF tau microtubule-binding region identifies pathological changes in primary tauopathies

**DOI:** 10.1038/s41591-022-02075-9

**Published:** 2022-11-24

**Authors:** Kanta Horie, Nicolas R. Barthélemy, Salvatore Spina, Lawren VandeVrede, Yingxin He, Ross W. Paterson, Brenton A. Wright, Gregory S. Day, Albert A. Davis, Celeste M. Karch, William W. Seeley, Richard J. Perrin, Rama K. Koppisetti, Faris Shaikh, Argentina Lario Lago, Hilary W. Heuer, Nupur Ghoshal, Audrey Gabelle, Bruce L. Miller, Adam L. Boxer, Randall J. Bateman, Chihiro Sato

**Affiliations:** 1 Department of Neurology, Washington University School of Medicine, St. Louis, MO USA; 2 The Tracy Family Stable Isotope Labeling Quantitation Center, Washington University School of Medicine, St. Louis, MO USA; 3 Department of Neurology, University of California San Francisco, San Francisco, CA USA; 4 Department of Neurology, University College London Queen Square Institute of Neurology, University College London, London, UK; 5 Department of Neurosciences, University of California San Diego School of Medicine, La Jolla, CA USA; 6 Department of Neurology, Mayo Clinic Florida, Jacksonville, FL USA; 7 Hope Center for Neurological Disorders, St. Louis, MO USA; 8 Department of Psychiatry, Washington University School of Medicine, St. Louis, MO USA; 9 Charles F. and Joanne Knight Alzheimer Disease Research Center, Washington University School of Medicine, St. Louis, MO USA; 10 Department of Pathology and Immunology, Washington University School of Medicine, St. Louis, MO USA; 11 Memory Research and Resources Center, Department of Neurology, University Hospital of Montpellier, Neurosciences Institute of Montpellier, University of Montpellier, Montpellier, France

**Keywords:** Neurodegenerative diseases, Diagnostic markers

## Abstract

Despite recent advances in fluid biomarker research in Alzheimer’s disease (AD), there are no fluid biomarkers or imaging tracers with utility for diagnosis and/or theragnosis available for other tauopathies. Using immunoprecipitation and mass spectrometry, we show that 4 repeat (4R) isoform-specific tau species from microtubule-binding region (MTBR-tau_275_ and MTBR-tau_282_) increase in the brains of corticobasal degeneration (CBD), progressive supranuclear palsy (PSP), frontotemporal lobar degeneration (FTLD)-*MAPT* and AD but decrease inversely in the cerebrospinal fluid (CSF) of CBD, FTLD-*MAPT* and AD compared to control and other FTLD-tau (for example, Pick’s disease). CSF MTBR-tau measures are reproducible in repeated lumbar punctures and can be used to distinguish CBD from control (receiver operating characteristic area under the curve (AUC) = 0.889) and other FTLD-tau, such as PSP (AUC = 0.886). CSF MTBR-tau_275_ and MTBR-tau_282_ may represent the first affirmative biomarkers to aid in the diagnosis of primary tauopathies and facilitate clinical trial designs.

## Abstract (web summary)

Cerebrospinal fluid measures of isoform-specific tau species from the microtubule-binding region serve as the first fluid biomarkers of primary tauopathy.

## Main

Tauopathies are a heterogeneous group of neurodegenerative diseases that all include aggregated tau proteins. The symptomatic phases of these fatal illnesses involve neurological impairments that typically progress over years to decades, leading to substantial medical, social and financial burden on patients and families. For the most common tauopathy, AD, the CSF biomarkers amyloid-β (Aβ) and total and phosphorylated tau have been used to aid diagnosis^1–8^ and these biomarkers are useful for assessing the outcome of therapies in clinical trials^9–11^. Additionally, recent progress in positron emission tomography (PET) imaging now enables the measurement of aggregated Aβ and tau in the brains of living patients with AD^12^. In contrast, such progress is lacking for other tauopathies classified as FTLD-tau, including CBD, PSP, argyrophilic grain disease (AGD), globular glial tauopathy, chronic traumatic encephalopathy (CTE) and Pick’s disease (PiD). Accurate diagnoses are challenging without fluid biomarkers for the tauopathies because these disorders fall within a spectrum comprising multiple and overlapping clinical phenotypes. Most tauopathies can only be definitively diagnosed by brain autopsy. Antemortem fluid biomarkers for these tauopathies will be required to improve the accuracy of clinical diagnosis and facilitate clinical trials for tauopathy therapeutics.

Recent structural and biochemical analyses implicate that distinct tau species constitute brain tau aggregates in subtypes of tauopathies. Six tau splicing isoforms are expressed in the adult human brain, including isoforms containing R1, R3 and R4 (3R) and R1, R2, R3 and R4 (4R) repeat domains in the MTBR^13^. Tauopathies can be classified into 3R, 4R and 3R/4R mixed tauopathies based on the dominant isoforms found in tau aggregates. Cryogenic electron microscopy (cryo-EM) studies demonstrated that there are distinct tau filament structures in AD (3R/4R)^14^, PiD (3R)^15^, CBD (4R)^16^, PSP (4R)^17^ and CTE (3R/4R)^18^. R3 and R4 repeat domains are commonly present in tau aggregates from AD and other tauopathies^14^. In contrast, a 4R isoform-specific R2 repeat domain in addition to R3 and R4 are found in tau aggregates from CBD and PSP^16,17^. Recently, we used biochemical extraction and mass spectrometry (MS) to show that specific tau fragments, such as the residues 243–254 (MTBR-tau_243_; R1), 299–317 (MTBR-tau_299_; R2-R3) and 354–369 (MTBR-tau_354_; R4), differentially enriched in AD brains with disease progression^19^. Furthermore, we showed that the truncated tau containing MTBR could be detected and quantified in CSF and that CSF soluble concentrations of MTBR-tau fragments reflected AD clinical severity and correlated strongly with tau PET measures^19^. Others also reported a correlation between CSF soluble truncated MTBR-tau and insoluble tau aggregates measured by tau PET measures^20^, suggesting that measures of MTBR-tau fragments might serve as fluid biomarkers of tau aggregation in AD.

In this study, we hypothesized that 4R isoform-specific MTBR-tau species accumulate in the brain of specific subtypes of 4R tauopathies. Then, we investigated if these changes are reflected in the CSF and can distinguish different subtypes of primary tauopathies. We specifically monitored tau fragments 275–280 (MTBR-tau_275_) and 282–290 (MTBR-tau_282_) that are in the R2 region and specific to 4R tau splicing isoforms. We tested differential diagnostic abilities of MTBR-tau_275_ and MTBR-tau_282_ normalized to total tau, to distinguish FTLD-tau, FTLD with TAR DNA-binding protein aggregates (FTLD-TDP), control and within different subtypes of tauopathies.

## Results

### 4R-specific brain MTBR-tau increases in primary tauopathies

**Fig. 1:**
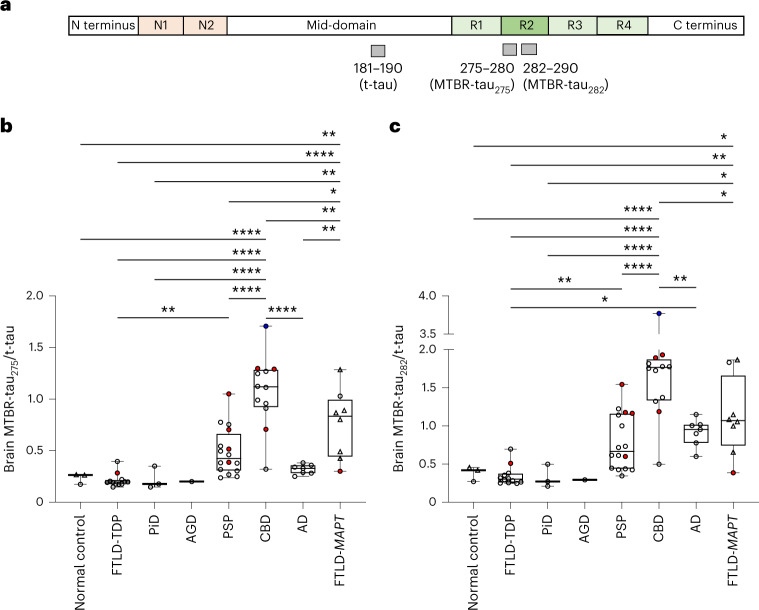
4R-specific insoluble brain MTBR-tau is enriched in CBD, FTLD-*MAPT*, AD and PSP. **a**, Schematic of the quantified peptides of t-tau_181–190_ and 4R isoform-specific MTBR-tau in the R2 region (gray bars, MTBR-tau_275_ and MTBR-tau_282_). The relative abundance of each MTBR-tau was normalized to the t-tau peptide. **b**,**c**, MTBR-tau_275_/t-tau (**b**) and MTBR-tau_282_/t-tau (**c**) were measured in the tauopathy patient’s insoluble brain fractions from the SFG (circle, *n* = 54) and insula (triangle, *n* = 8). Both MTBR-tau species were most enriched in CBD (*n* = 12) and FTLD-*MAPT* (*n* = 8). PSP (*n* = 16) and AD (*n* = 7) had moderate enrichment. AGD (*n* = 1), PiD (*n* = 3) and FTLD-TDP (*n* = 12) did not change in MTBR-tau_275_ or MTBR-tau_282_ compared to normal control (*n* = 3). The red (*n* = 9) and blue (*n* = 1) filled circles indicate AD and PSP copathology, respectively. *P < 0.05, **P < 0.01, ****P < 0.0001. The box plots show the minimum, 25th percentile, median, 75th percentile and maximum. Differences in biomarker values were assessed with a one-way ANOVA. A two-sided *P* < 0.05 was considered statistically significant and corrected for multiple comparisons using a Benjamini–Hochberg FDR set at 5%.

First, frozen brain tissues from 59 individuals with autopsy-confirmed AD, FTLD-tau or FTLD-TDP and three normal control individuals without clinical diagnoses of neurodegenerative diseases were processed for biochemical extraction of insoluble tau and underwent MS analyses (Table 1). The superior frontal gyrus (SFG) at the level of the frontal eye fields and the insular cortex were chosen as regions of interests because of the higher severity of tau aggregation seen in these regions across the clinical spectrum of primary tauopathies ranging from behavioral variant frontotemporal dementia (bvFTD) to Richardson’s syndrome^21^. MTBR-tau_275_ (275–280) and MTBR-tau_282_ (282–290) located within the R2 repeat domain and specific to 4R isoforms were normalized with total tau (t-tau) measured by mid-domain tau peptide 181–190 to account for individual variabilities in t-tau (Fig. 1a). Brain MTBR-tau_275_/t-tau increased approximately fivefold in CBD (1.078 ± 0.346) and threefold in FTLD*-MAPT* (0.762 ± 0.333) compared to normal control (0.235 ± 0.053, *P* < 0.0001, *P* < 0.0001, respectively) and non-tauopathy FTLD-TDP (0.213 ± 0.066, *P* < 0.0001, *P* < 0.01, respectively; Fig. 1b). Interestingly, MTBR-tau_275_/t-tau increased in CBD and FTLD*-MAPT* compared to other tauopathies, such as 3R tauopathy, PiD (0.226 ± 0.110, *P* < 0.0001, *P* < 0.01, respectively), 4R tauopathies, AGD (0.200) and PSP (0.489 ± 0.229, *P* < 0.0001, *P* < 0.05, respectively), and 3R/4R mixed tauopathy, AD (0.319 ± 0.047, *P* < 0.0001, *P* < 0.01, respectively). MTBR-tau_275_/t-tau moderately increased in PSP (approximately twofold) compared to FTLD-TDP (*P* < 0.01). Brain MTBR-tau_282_/t-tau had a similar increase profile to MTBR-tau_275_/t-tau (Fig. 1c) but moderately (2.6-fold) increased in AD (0.915 ± 0.180) compared to FTLD-TDP (0.348 ± 0.133, *P* < 0.05), which was not observed in MTBR-tau_275_/t-tau. These results suggest that 4R-specific MTBR-tau species are enriched in the insoluble fraction of SFG/insular cortex brain tissue in a subset of 4R tauopathies, such as CBD and FTLD*-MAPT*, and moderately increased in a different PSP (4R tauopathy) and AD (3R/4R mixed tauopathy).

**Table 1:** Demographics and brain MTBR-tau measures of participants in the primary tauopathy cohort

Group	Normal control	FTLD-TDP	PiD	AGD	PSP	CBD	AD	FTLD*-MAPT*	Total
*n*	3	12	3	1	16	12	7	8	62
Pure pathology	N/A	5	3	0	9	7	7	6	37
Copathology (*n*)	N/A	AD (1), AGD (1), HS + vascular (1), MND (4)	N/A	Vascular (1)	AD (4), LBD (2), vascular (1)	AD (3), PSP (1), LBD (1)	N/A	AD (1), HS (1)	22
Age at death, years	81 ± 5	68 ± 11	70 ± 3	68	76 ± 7	69 ± 8	61 ± 6	63 ± 11	69 ± 10
Sex, M/F	1/2	8/4	1/2	1/0	6/10	5/7	4/3	5/3	31/31
Brain region (SFG/insula)	1/2	12/0	3/0	1/0	16/0	12/0	7/0	2/6	54/8
Brain MTBR-tau_275_/t-tau, SD	0.235 ± 0.053	0.213 ± 0.066	0.226 ± 0.110	0.200	0.489 ± 0.229	1.078 ± 0.346	0.319 ± 0.047	0.762 ± 0.333	0.536 ± 0.388
Brain MTBR-tau_282_/t-tau, SD	0.382 ± 0.097	0.348 ± 0.133	0.327 ± 0.151	0.294	0.789 ± 0.368	1.736 ± 0.760	0.915 ± 0.180	1.128 ± 0.512	0.895 ± 0.646

HS, hippocampal sclerosis; LBD, Lewy body dementia; MND, motor neuron disease.

### 4R-specific CSF MTBR-tau decreases in primary tauopathies

**Fig. 2:**
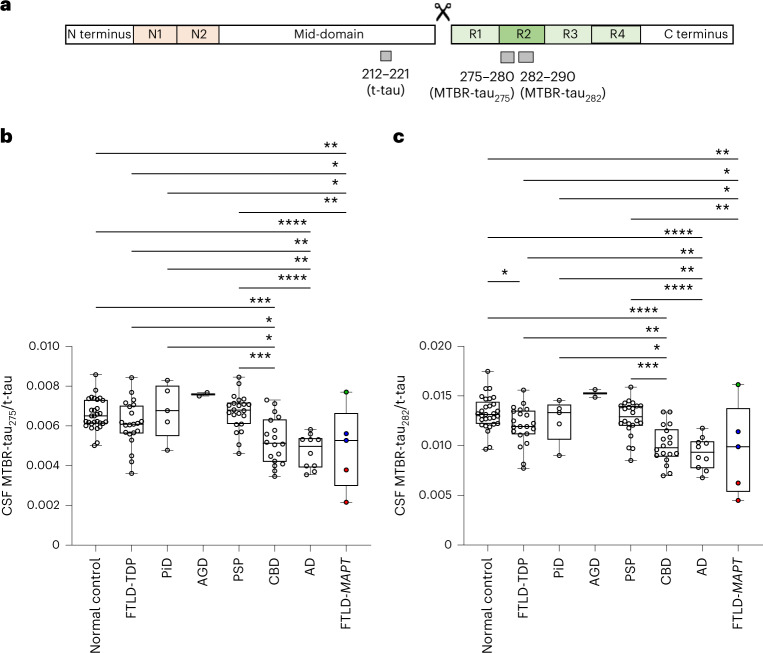
4R-specific CSF MTBR-tau decreases in CBD, FTLD-*MAPT* and AD. **a**, Schematic of the quantified peptides of t-tau 212–221, truncation and 4R isoform-specific MTBR-tau in the R2 region (gray bars, MTBR-tau_275_ and MTBR-tau_282_). The relative abundance of each MTBR-tau was normalized to the t-tau peptide. **b**,**c**, CSF MTBR-tau_275_/t-tau (**b**) and MTBR-tau_282_/t-tau (**c**) significantly decreased in CBD (*n* = 18), AD (*n* = 10) and FTLD*-MAPT* (*n* = 5) compared to normal control (*n* = 29), FTLD-TDP (*n* = 21) and other FTLD-tau. FTLD*-MAPT* P301L (red, *n* = 2), R406W (blue, *n* = 2) and S305I (green, *n* = 1) decreased in MTBR-tau/t-tau measurements in this order. *P < 0.05, **P *<* 0.01, ***P < 0.001, ****P < 0.0001. The box plots show the minimum, 25th percentile, median, 75th percentile and maximum. Differences in biomarker values were assessed with a one-way ANOVA. A two-sided *P* < 0.05 was considered statistically significant and corrected for multiple comparisons using a Benjamini–Hochberg FDR set at 5%.

Next, CSF from 29 normal controls, 5 FTLD-*MAPT* and 78 autopsy-confirmed cases of AD, primary tauopathies and FTLD-TDP were analyzed for MTBR-tau_275_ and MTBR-tau_282_ (Table 2). CSF MTBR-tau_275_ and MTBR-tau_282_ concentrations did not separate different tauopathies (Supplementary Fig. 1a,b) due to individual variability in t-tau concentrations (Supplementary Fig. 1c). Therefore, CSF MTBR-tau_275_ and MTBR-tau_282_ from truncated tau were normalized by t-tau measured by mid-domain tau 212–221 (Fig. 2a), like the normalization methods previously reported in truncated tau and Aβ isoform measurements^20,22^. CSF MTBR-tau_275_/t-tau decreased in CBD (0.00525 ± 0.00117), AD (0.00472 ± 0.00085) and FTLD*-MAPT* (0.00491 ± 0.00207), compared to normal control (0.00657 ± 0.00078, *P* < 0.001, *P* < 0.0001, *P* < 0.01, respectively) and non-tauopathy control, FTLD-TDP (0.00611 ± 0.00115, *P* < 0.05, *P* < 0.01, *P* < 0.05, respectively; Figure 2b). This decrease was particularly substantial in FTLD-*MAPT* P301L, which has more typical FTLD pathology than in R406W, which has many features of AD. CSF MTBR-tau_275_ also decreased in CBD, AD and FTLD*-MAPT* compared to other 4R tauopathies, AGD (0.00759 ± 0.00013) and PSP (0.00669 ± 0.00091, *P* < 0.001, *P* < 0.0001, *P* < 0.01, respectively), and the 3R tauopathy, PiD (0.00676 ± 0.00138, *P* < 0.05, *P* < 0.01, *P* < 0.05, respectively). CSF MTBR-tau_282_/t-tau had similar decrease profiles to CSF MTBR-tau_275_/t-tau (Fig. 2c). Interestingly, CSF MTBR-tau_275_/t-tau did not change in PSP compared to control or FTLD-TDP even though these ratios increased moderately in the brain.

**Table 2:** Demographics and CSF MTBR-tau measures of participants in the pathologically confirmed primary tauopathy cohort

Group	Normal control^a^	FTLD-TDP^a^	PiD^a^	AGD^a^	PSP^a^	CBD^a^	AD^a^	FTLD-*MAPT*^a^	Total
***n***	29	21	5	2	22	18	10	5	112
**Autopsy-confirmed**	1	21	5	2	22	18	10	3	82
**Pure pathology**	N/A	14	5	1	14	13	10	1	58
**Copathology (*n*)**	N/A	AD (1), AGD (1), HS + vascular (1), MND (4)	N/A	Vascular (1)	AD (5), LBD (2), vascular (1)	AD (3), PSP (1), LBD (1)	N/A	AD (1), HS (1)	23
**Clinical syndromes**	N/A	ALS + MCI (1), MCI (1), bvFTD (7), bvFTD + ALS (6), CBS (1), DLB (1), nfvPPA (1), nfvPPA + ALS (1), PPS (1), svPPA (1)	bvFTD (3), nfvPPA (2)	bvFTD (2)	CBS (5), nfvPPA (5), PAGF (1), PSP-RS (11)	bvFTD (4), CBS (9), nfvPPA (4), PSP-RS (1)	AD (1), AD + TES (1), EOAD (3), lvPPA (1), PCA (4)	Cognitively normal (2), bvFTD (2), nfvPPA (1)	83
**Age at onset, SD**	N/A	60 ± 10	59 ± 4	53 ± 11	65 ± 7	60 ± 7	53 ± 4	49 ± 12 (3)	60 ± 9 (81)
**Age at CSF sampling, SD**	62 ± 13	64 ± 9	64 ± 9	68 ± 1	70 ± 7	64 ± 7	57 ± 5	50 ± 12	64 ± 10
**Interval between CSF collection and death, SD**	2 (1)	2 ± 2	5 ± 2	0 ± 0	3 ± 2	3 ± 2	4 ± 1	2 ± 1 (3)	3 ± 2 (82)
**Duration, SD**	N/A	4 ± 4	6 ± 2	15 ± 11	5 ± 3	4 ± 1	5 ± 3	5 ± 5 (3)	5 ± 4 (81)
**Sex, M/F**	14/15	12/9	3/2	2/0	10/12	8/10	6/4	2/3	57/55
**CDR plus NACC FTLD SB score**	0.2 ± 0.5 (17)	10.6 ± 5.1 (17)	11.0 ± 2.1 (5)	9.3 ± 3.2 (2)	5.3 ± 3.1 (13)	6.7 ± 3.7 (17)	6.6 ± 2.2 (7)	6.6 ± 6.3 (5)	6.2 ± 5.0 (83)
**CSF MTBR-tau**_**275**_**/t-tau, SD**	0.00657 ± 0.00078	0.00611 ± 0.00115	0.00676 ± 0.00138	0.00759 ± 0.00013	0.00669 ± 0.00091	0.00525 ± 0.00117	0.00472 ± 0.00085	0.00491 ± 0.00207	0.00608 ± 0.00126
**CSF MTBR-tau**_**282**_**/t-tau, SD**	0.01328 ± 0.00167	0.01203 ± 0.00193	0.01256 ± 0.00216	0.01526 ± 0.00054	0.01273 ± 0.00179	0.01007 ± 0.00191	0.00925 ± 0.00161	0.00963 ± 0.00457	0.01190 ± 0.00244

^a^Numbers in brackets indicate the number of available information within the group if limited. ALS, amyotrophic lateral sclerosis; DLB, dementia with Lewy bodies; EOAD, early-onset AD; lvPPA, logopenic variant of PPA; MCI: mild cognitive impairment; PAGF, pure akinesia with gait freezing; PCA, posterior cortical atrophy; PPS, pallidopyramidal syndrome; svPPA: semantic variant of PPA; TES, traumatic encephalopathy syndrome.

**Fig. 3:**
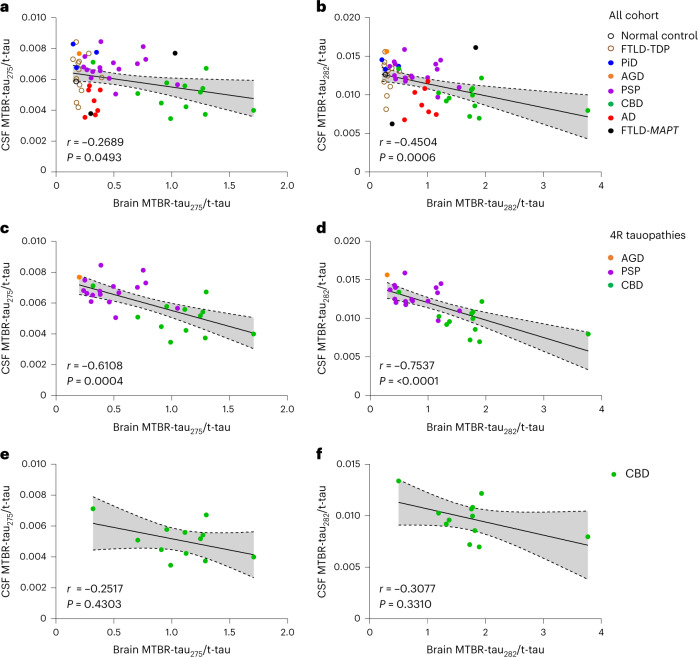
CSF soluble MTBR-tau correlates with brain insoluble MTBR-tau aggregates. **a**,**b**, MTBR-tau_275_/t-tau (**a**) and MTBR-tau_282_/t-tau (**b**) from paired CSF and brain inversely correlated in tauopathies, FTLD-TDP and control (*n* = 54, *r* = −0.27, *P* = 0.049, −0.45, *P* = 0.0006, respectively). **c**,**d**, MTBR-tau_275_/t-tau (**c**) and MTBR-tau_282_/t-tau (**d**) from paired CSF and brain had higher correlations (*r* = −0.61, *P* = 0.0004 and *r* = −0.75, *P* < 0.0001, respectively) in 4R tauopathies (CBD, PSP and AGD, *n* = 29). **e**,**f**, MTBR-tau_275_/t-tau (**e**) and MTBR-tau_282_/t-tau (**f**) from paired CSF and brain correlated in CBD (*n* = 12, *r* = −0.25, *P* = 0.43 and *r* = −0.31, *P* = 0.33, respectively). The gray shadow represents the 95% confidence intervals for the linear regression.

To assess if the soluble CSF MTBR-tau/t-tau measures reflected brain tau pathology measured by the paired insoluble brain MTBR-tau/t-tau measures, MTBR-tau/t-tau from antemortem CSF and brain from the same individuals were analyzed for correlation (*n* = 54; Fig. 3a,b). MTBR-tau_275_/t-tau and MTBR-tau_282_/t-tau from the CSF and brain correlated moderately (*r* = −0.27, *P* = 0.049 and *r* = −0.45, *P* = 0.0006, respectively) across all disease groups (*n* = 54) and strongly (*r* = −0.61, *P* = 0.0004 and *r* = 0.75, *P* < 0.0001; Fig. 3c,d) in 4R tauopathies (PSP, CBD and AGD, *n* = 29). This suggests that 4R-specific MTBR-tau species have inverse correlation in the CSF and brain in 4R tauopathies. In CBD, MTBR-tau_275_/t-tau and MTBR-tau_282_/t-tau from the CSF and brain correlated moderately but no statistical significance was obtained (*n* = 12, *r* = −0.25, *P* = 0.43 and *r* = −0.31, *P* = 0.33, respectively; Figure 3e,f). One CBD participant who had no cognitive impairment (Clinical Dementia Rating plus National Alzheimer’s Coordinating Center FTLD sum of boxes^23^ (CDR plus NACC FTLD-SB) = 0) had fewer changes in MTBR-tau_275_/t-tau and MTBR-tau_282_/t-tau in both brain and CSF (brain MTBR-tau_275_/t-tau = 0.321, brain MTBR-tau_282_/t-tau = 0.499, CSF MTBR-tau_275_/t-tau = 0.0071, CSF MTBR-tau_282_/t-tau = 0.0134). This may suggest that the changes of these biomarkers depend on the severity of the disease.

To assess if CSF MTBR-tau_275_/t-tau and MTBR-tau_282_/t-tau decrease with disease stage, the correlations between these biomarkers and duration (the interval between age at onset and CSF collection) were investigated (Table 2 and Supplementary Fig. 2). Average duration across diseases was 5 ± 4 years (*n* = 81) and 4 ± 1 years for CBD-only (*n* = 18). In CBD, there were negative correlations between duration and CSF MTBR-tau_275_/t-tau and MTBR-tau_282_/t-tau (*r* = −0.37 and −0.39, respectively) although statistical significance was not observed (*P* = 0.13 and 0.11, respectively). This result suggests that participants with longer duration who are at later pathological stages have larger degrees of decrease in the CSF MTBR-tau_275_/t-tau and MTBR-tau_282_/t-tau biomarkers.

### CSF MTBR-tau is reproducible in repeated lumbar punctures

**Extended Data Fig. 1:**
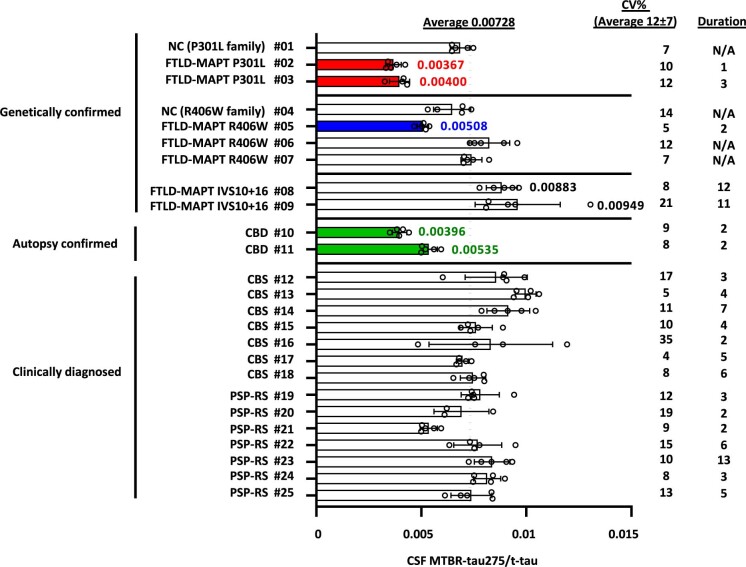
4R CSF MTBR-tau assay is reproducible and stable in repeated lumbar punctures. CSF MTBR-tau_275_/t-tau was measured in 25 participants (#01-#25) who had 3 to 5 repeated lumbar punctures within 4 months. FTLD*-*MAPT P301L (red, n = 2, #02, 03), symptomatic FTLD*-*MAPT R406W (blue, n = 1, #05), and pathologically-confirmed CBD (green, n = 2, #10 and #11) had decreased CSF MTBR-tau_275_/t-tau. Data are presented as mean values + /− SD. CBS: corticobasal syndrome, PSP-RS: progressive supranuclear palsy-Richardson’s syndrome, FTLD*-*MAPT: frontotemporal lobar degeneration with *MAPT* mutations.

To evaluate reproducibility and stability of the CSF MTBR-tau measurements within the same individual, we examined CSF MTBR-tau_275_/t-tau in an independent cohort of 25 participants who underwent repeated lumbar punctures (LPs) (3–5 times) within approximately 4 months as a part of an ongoing study examining protein turnover kinetics^24^ (Extended Data Table 1 and Extended Data Fig. 1). These participants include individuals clinically diagnosed with PSP-Richardson’s syndrome (PSP-RS, *n* = 7) or corticobasal syndrome (CBS, a clinical syndrome associated with heterogenous neuropathological substrates including AD, CBD, PSP and FTLD-TDP; *n* = 9), with two participants having autopsy-confirmed CBD. They also included seven *MAPT* mutation (P301L, R406W and IVS10+16) carriers who were either symptomatic or asymptomatic and two noncarrier family members who are normal controls. The mean coefficient of variation for CSF MTBR-tau_275_/t-tau in repeated LPs was 12 ± 7%, establishing the high reproducibility and stability of CSF MTBR-tau measures within 4 months.

Consistent with the FTLD-*MAPT* cases analyzed with the pathologically confirmed cohort, CSF MTBR-tau_275_/t-tau decreased in FTLD-*MAPT* mutation carriers in the repeated LP cohort. Interestingly, CSF MTBR-tau_275_/t-tau decreased in two symptomatic FTLD-*MAPT* P301L mutation carriers (participant numbers 02 and 03, 0.00381 ± 0.00021) and a symptomatic FTLD-*MAPT* R406W mutation carrier (number 05, 0.00508) compared to normal control (numbers 01 and 04, 0.00666 ± 0.00027). However, CSF MTBR-tau_275_/t-tau did not change in FTLD-*MAPT* R406W mutation carriers who were asymptomatic at LPs (numbers 06 and 07). The FTLD*-MAPT* variant IVS10+16 promotes the splicing of tau exon 10, resulting in greater production of 4R over 3R isoforms. Indeed, symptomatic FTLD*-MAPT* IVS10+16 mutation carriers (numbers 08 and 09, 0.00921 ± 0.00053) had 1.38-fold higher CSF MTBR-tau_275_/t-tau compared to normal controls, indicating that an increase in 4R isoforms is reflected in the CSF.

CSF MTBR-tau_275_/t-tau decreased in the two participants who were clinically diagnosed as PSP-RS but later were autopsy-confirmed with CBD (numbers 10 (0.00396) and 11 (0.00535)), which is consistent with the pathologically confirmed CSF cohort results. However, CSF MTBR-tau_275_/t-tau did not change in participants clinically diagnosed with PSP-RS (0.00779 ± 0.00052) or CBS (0.00748 ± 0.00187), who are not yet autopsy-confirmed during the repeated measures studies. Average duration across diseases was 5 ± 3 years (*n* = 21) and 4 ± 2 for CBS only (*n* = 9), which were similar to the pathologically confirmed cohort.

### CSF MTBR-tau in clinically diagnosed primary tauopathies

**Extended Data Fig. 2:**
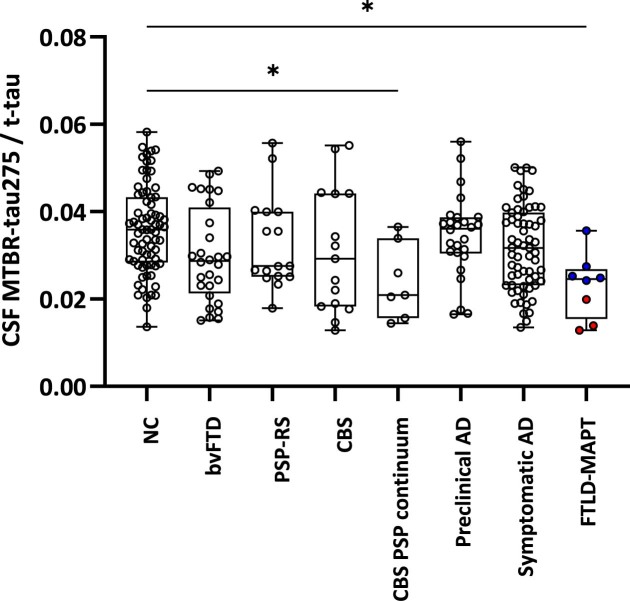
CSF MTBR-tau_275_/t-tau decreases in clinically diagnosed CBS-PSP continuum and FTLD*-MAPT*. CSF MTBR-tau_275_/t-tau decreased in clinically diagnosed CBS-PSP continuum (n = 7), and genetically confirmed FTLD*-*MAPT (red: n = 3, P301L, blue: n = 5, R406W) compared to NC (n = 88). Differences in biomarker values were assessed with one-way ANOVAs. A two-sided p < 0.05 was considered statistically significant and corrected for multiple comparisons using Benjamini-Hochberg false discovery rate (FDR) method with FDR set at 5%. *P < 0.05. The box plots show the minimum, 25 percentile, median, 75 percentile, and maximum. NC: normal control, bvFTD: behavioral variant frontotemporal dementia (n = 28), PSP-RS: progressive supranuclear palsy-Richardson’s syndrome (n = 16), CBS: corticobasal syndrome (n = 15), AD: Alzheimer’s disease (n = 80), FTLD*-*MAPT*:* frontotemporal lobar degeneration with *MAPT* mutations (n = 8).

To estimate the CSF MTBR-tau biomarker performance in clinically diagnosed primary tauopathies, we measured CSF MTBR-tau_275_/t-tau in an additional independent cohort of 238 primary tauopathies with single LP (Extended Data Fig. 2). This cohort was previously analyzed for CSF t-tau and phosphorylated tau^25^ and includes clinically diagnosed cases of AD, sporadic bvFTD, bvFTD secondary to FTLD*-MAPT*, PSP-RS, CBS and the CBS-PSP continuum^26^. Individuals with the CBS-PSP continuum are defined as patients who initially presented with CBS but subsequently developed clinical features of PSP-RS as the disease progressed. CSF MTBR-tau_275_/t-tau decreased in the CBS-PSP continuum and FTLD*-MAPT* compared to cognitively normal controls (*P* < 0.05). However, CSF MTBR-tau_275_/t-tau did not statistically change in either AD or clinically diagnosed CBS compared to control or other tauopathies.

### Diagnostic accuracies of CSF MTBR-tau in primary tauopathies

Finally, we examined the diagnostic accuracies of CSF MTBR-tau_275_/t-tau and MTBR-tau_282_/t-tau in a pathologically confirmed primary tauopathy cohort. First, CSF t-tau (mid-domain peptide 212–221) and phosphorylated tau (pT217/T217) were examined in primary tauopathies for comparison (Supplementary Figs. 1c and 3a). CSF t-tau increased in AD compared to normal control and PSP (*P* < 0.05) and can differentiate AD from FTLD-tau (PSP, CBD, AGD, PiD, FTLD-*MAPT)* with an AUC of 0.794 (Supplementary Fig. 3b). However, CSF t-tau does not distinguish among FTLD-tau. CSF pT217/T217 increased in AD compared to normal control, FTLD-TDP and FTLD-tau (*P* < 0.0001) and can differentiate AD from FTLD-tau with an AUC of 0.987 (Supplementary Fig. 3c). AD copathology in other neurodegenerative diseases (for example, FTLD-TDP, CBD, PSP) also increased CSF pT217/T217. These results suggest that we can use CSF pT217/T217 to accurately identify individuals with AD pathology, regardless of copathology.

Effect of amyloid on CSF MTBR-tau_275_/t-tau and MTBR-tau_282_/t-tau in primary tauopathies were further assessed using AD Thal phase. CSF pT217/T217 strongly correlated with AD Thal phase (*r* = 0.52, *P* < 0.0001; Supplementary Fig. 4a). However, CSF MTBR-tau_275_/t-tau and MTBR-tau_282_/t-tau did not correlate with AD Thal phase in the whole cohort (*r* = −0.22, *P* = 0.06 and *r* = −0.24, *P* = 0.04, respectively; Supplementary Fig. 4b,c) or in CBD (*r* = −0.14, *P* = 0.60 and *r* = −0.07, *P* = 0.78, respectively; Supplementary Fig. 4d,e). These results suggest that CSF MTBR-tau_275_/t-tau and MTBR-tau_282_/t-tau decreased in CBD independently from AD copathology.

The diagnostic accuracies of CSF MTBR-tau_275_/t-tau and MTBR-tau_282_/t-tau were examined to determine if we can distinguish CBD from control, FTLD-TDP and FTLD-tau as a group and individual tauopathy (Table 3 and Supplementary Fig. 5). CSF MTBR-tau_275_/t-tau and CSF MTBR-tau_282_/t-tau can distinguish CBD from normal control, other FTLD-tau (PSP, PiD and AGD), PiD and PSP with AUCs of 0.800–0.889. CBD can be distinguished from FTLD-TDP with AUCs of 0.701–0.770. When AD copathology cases were excluded, CSF MTBR-tau_275_/t-tau and CSF MTBR-tau_282_/t-tau can distinguish CBD from PSP with AUCs of 0.859 and 0.886, respectively (Table 3 and Supplementary Fig. 5k,l).

**Table 3:** Diagnostic accuracies of 4R-specific CSF MTBR-tau to distinguish CBD from FTLD-tau and control

Groups	*n* per group	Test	AUC	95% CI	*P*	Sensitivity (%)	Specificity (%)	Cutoff
CBD versus normal control	18 versus 29	CSF MTBR-tau_275_/t-tau	0.810	0.672–0.949	0.0004	93.1	72.2	0.00582
		CSF MTBR-tau_282_/t-tau	0.889	0.790–0.988	<0.0001	89.7	77.9	0.01170
CBD versus FTLD-TDP	18 versus 21	CSF MTBR-tau_275_/t-tau	0.701	0.530–0.872	0.0323	81.0	66.7	0.00559
		CSF MTBR-tau_282_/t-tau	0.770	0.619–0.921	0.0041	81.0	72.2	0.01096
CBD versus other FTLD-tau (PSP, AGD, PiD)	18 versus 29	CSF MTBR-tau_275_/t-tau	0.835	0.716–0.955	0.0001	89.7	66.7	0.00563
		CSF MTBR-tau_282_/t-tau	0.855	0.747–0.963	<0.0001	75.9	89.0	0.01219
CBD versus PiD	18 versus 5	CSF MTBR-tau_275_/t-tau	0.800	0.572–1.000	0.0442	80.0	72.2	0.00599
		CSF MTBR-tau_282_/t-tau	0.806	0.543–1.000	0.0404	80.0	89.0	0.01219
CBD versus PSP	18 versus 22	CSF MTBR-tau_275_/t-tau	0.828	0.696–0.960	0.0004	90.0	66.7	0.00563
		CSF MTBR-tau_282_/t-tau	0.854	0.736–0.972	0.0001	72.7	89.0	0.01220
CBD versus PSP (AD copathology removed)	15 versus 17	CSF MTBR-tau_275_/t-tau	0.859	0.718–0.999	0.0005	82.4	86.7	0.00648
		CSF MTBR-tau_282_/t-tau	0.886	0.763–1.000	0.0002	94.1	80.0	0.01164

CI, confidence interval.

Lastly, we retrospectively assessed CSF MTBR-tau_275_/t-tau and MTBR-tau_282_/t-tau by final clinical syndromes in a neuropathologically confirmed cohort to determine if these biomarkers can facilitate antemortem diagnosis of primary tauopathies. The numbers of individuals who had CSF MTBR-tau_275_/t-tau and MTBR-tau_282_/t-tau lower than cutoff (0.00563 and 0.01220, respectively; defined in Table 3 to differentiate CBD and PSP) within each clinical syndrome and neuropathological diagnosis are summarized (Extended Data Tables 2 and 3 and Supplementary Fig. 6). CBD (*n* = 18) had clinical syndromes of either CBS (*n* = 9), bvFTD (*n* = 4), nonfluent variant PPA (nfvPPA, *n* = 4) or PSP-RS (*n* = 1). Without biomarkers, the diagnostic accuracy of CBD within CBS was 9 out of 15 (60%). Among 15 individuals with CBS, 7 and 10 had lower than cutoff values of CSF MTBR-tau_275_/t-tau and MTBR-tau_282_/t-tau and 6 and 7 were CBD, respectively. With these biomarkers, the diagnostic accuracies of CBD within CBS were 6 out of 7 (86%) and 7 out of 10 (70%) for CSF MTBR-tau_275_/t-tau and MTBR-tau_282_/t-tau, respectively. Across different clinical syndromes of CBD, 12 out of 18 (67%) and 15 out of 18 (83%) had CSF MTBR-tau_275_/t-tau and MTBR-tau_282_/t-tau values lower than cutoff, suggesting that these biomarkers identify CBD regardless of clinical syndromes with up to 83% accuracy. Other applications of these biomarkers include distinguishing CBD from PSP among PSP-RS since 0 out of 11 (0%) and 2 out of 11 (18%) PSP had lower than cutoff for CSF MTBR-tau_275_/t-tau and MTBR-tau_282_/t-tau, respectively.

## Discussion

Despite the long quest for antemortem biomarkers of FTLD-tau pathology, to date no fluid biomarkers have been identified that can differentiate subgroups of tauopathies other than AD. Previous studies showed that CSF phosphorylated tau at T181 decreased in PSP and FTLD-TDP relative to controls^27–31^. We recently demonstrated that CSF pT217/T217 increased in FTLD-*MAPT* R406W compared to control without Aβ pathology^25^. However, phosphorylated tau changes most substantially in AD and thus these studies may not have captured key pathological changes in primary tauopathies. In this study, we focused on MTBR-tau, which constitutes the core regions of tau aggregates in the brain and also exists in the CSF as a truncated C-terminal tau fragment^19^. Using biochemical purification and quantitative MS, we showed that 4R isoform-specific MTBR-tau_275_ and MTBR-tau_282_ that are normalized to t-tau decrease in the CSF soluble tau as they increase in brain insoluble tau in primary tauopathies, especially CBD and FTLD-*MAPT* P301L. MTBR-tau/t-tau measures are inversely correlated in the CSF and brain, suggesting that there may be an equilibrium or a unidirectional transfer between soluble CSF MTBR-tau and insoluble brain MTBR-tau in these primary tauopathies. This study provides the possibility of the first fluid biomarker that reflects brain pathology in primary tauopathies.

One of the interesting findings of this study was that changes in 4R isoform-specific MTBR-tau_275_ and MTBR-tau_282_ were only observed in a subset of 4R and 3R/4R mixed tauopathies. As expected, these 4R isoform-specific measures did not change in 3R tauopathy (PiD) or non-tauopathy FTLD (FTLD-TDP). However, CSF MTBR-tau_275_/t-tau and MTBR-tau_282_/t-tau specifically decreased in CBD and FTLD*-MAPT* but not PSP and AGD even though all are categorized as 4R tauopathies. This may be due to higher variability in the degree of neocortical pathology in PSP and AGD cases compared to CBD and most FTLD*-MAPT* cases. Many cases of PSP have 4R tau aggregates primarily in subcortical regions, such as the thalamus and brain stem^32^, and AGD pathology is most severe within the medial temporal lobe, even in advanced stages^33^. In contrast, CBD has more abundant and widespread tau pathology in the cerebrum^34^. FTLD*-MAPT* (for example, P301L) can lead to a very high deposition of 4R tau aggregates in neurons and glia in multiple brain regions, including the hippocampus, neocortex and substantia nigra^35^. An alternative explanation is that CBD has wispy and fine filamentous inclusions within neuronal cell bodies, whereas PSP neurons tends to harbor a larger proportion of more compact tau aggregates^36^. CBD is commonly associated with abundant cortical astrocytic plaque pathology and neuritic tau pathology in both gray and white matter, while PSP neuronal and astrocytic pathology (tufted astrocytes) are often restricted to the motor and premotor cortex and subcortical nuclei. It is possible that tau aggregates in PSP may have different physicochemical property from CBD and the status of equilibrium between insoluble and soluble forms may be different. Together, we speculate that the quantity or total burden of 4R tau pathology in the whole brain may reflect changes in CSF 4R-specific MTBR-tau.

CSF MTBR-tau_275_/t-tau and MTBR-tau_282_/t-tau may potentially be used to positively identify a subset of primary tauopathies and may be useful in assisting with antemortem differential diagnosis. We confirmed in our repeated lumbar puncture study that CSF MTBR-tau/t-tau measures are reproducible and stable over 4 months, which will reliably provide biomarker values in the clinic or clinical trial settings. In the clinically diagnosed cohorts without autopsy confirmation, CSF MTBR-tau_275_/t-tau did not change in CBS and there was a higher overlap between CBS and PSP-RS, which might be attributable to lack of one-to-one relationship between clinical syndromes and neuropathological diagnosis in FTLD. However, from retrospective clinical syndrome analyses in the pathologically confirmed cohort, we showed that CSF MTBR-tau_275_/t-tau and MTBR-tau_282_/t-tau biomarkers may be able to identify individuals with CBD regardless of clinical syndromes (for example, CBS, bvFTD and PSP-RS) with as high as 83% accuracy, which is higher than approximately 25–50% diagnostic accuracies of CBD without these biomarkers.

Additional limitations of this study include the following: tauopathies with shorter duration or during the asymptomatic stage may not yet show decrease in these biomarkers. Future studies targeting larger cohorts with different severity and longitudinal samples with clinical measures would help address whether CSF MTBR-tau can capture disease at an earlier stage of primary tauopathies; the lack of orthogonal measures to identify brain tau pathology in living patients (for example, tau PET imaging with a tracer specific to primary tauopathies) limited our ability to assess correlation between CSF and brain MTBR-tau antemortem; the moderate size of the cohorts with small sample sizes for subgroups such as AGD and PiD can limit interpretation; the 77G7 MTBR-tau antibody used for sequential immunoprecipitation in the study may also be targeting specific pools of truncated tau; and there may be future technical advancements and analytical method developments that may unveil additional or new populations of tau species in biofluids that reflect qualitative and quantitative aspects of tau pathology in primary tauopathies. Overall, these findings advance our knowledge of heterogeneous pathophysiology in primary tauopathies and open avenues for the development of therapeutics and clinical trials targeting primary tauopathies.

## Methods

### Human studies

The retrospective study of pathologically confirmed cohort included participants seen at UCSF or participating research sites under the following projects: Hillblom Healthy Aging Study; UCSF Alzheimer’s Disease Research Center Program Project Grant (P30AG062422); ARTFL-LEFFTDS Longitudinal Frontotemporal Lobar Degeneration (ALLFTD) (U19AG063911); and the Four Repeat Tauopathy Neuroimaging Initiative (R01AG038791). Collection and use of biospecimens was approved by the institutional review board at ALLFTD or each research center from which the individual was recruited and this study was approved by the Biospecimen Resource Committee at UCSF. Participants provided written informed consent at the time of recruitment. Participants underwent a standardized clinical evaluation that included collection of demographic data, structured participant/informant interview, functional assessment, neurological examination and neuropsychological testing. CSF draws were performed at the same visit as the clinical evaluation; primary clinical syndrome was determined based on all available data at the time of clinical evaluation by an experienced neurologist or panel of neurologists according to established diagnostic criteria. Consent to undergo autopsy was provided by the patient or their surrogate according to the principles outlined in the Declaration of Helsinki 2013.

The repeated LP study (NCT03545126) was approved by the institutional review board at Washington University in St. Louis and University College London. All participants or their delegates consented to the collection and sharing of biofluid samples and brain autopsy and were compensated. Exclusion criteria included any contraindications for LPs or lumbar catheters, including a bleeding disorder, active anticoagulation and active infection.

The study of the clinically diagnosed cohort was approved by the ethics committee of the Montpellier University Hospital (CSF-Neurobank no. DC-2008-417 at the certified NFS 96–900 CHU resource center BB-0033-00031 (http://www.biobanques.eu)). Authorization to handle personal data was granted by the French Data Protection Authority (Commission Nationale de l’Informatique et des Libertés) under no. 1709743 v0.

### Human brain samples

Neuropathological diagnoses of human brain donations were rendered according to established diagnostic criteria at Washington University and UCSF. The frozen human brain tissue samples selected for this study were processed as described previously^19,37^. Briefly, frozen brain tissues were sliced using a cryostat and sonicated in ice-cold buffer containing 25 mM Tris hydrochloride (pH 7.4), 150 mM sodium chloride, 10 mM EDTA, 10 mM EGTA, phosphatase inhibitor cocktail and protease inhibitor cocktail. The homogenate was clarified by centrifugation for 20 min at 11,000 *g* at 4 °C and stored at −80 °C as the whole-brain extract. The demographics of the brain donors included in this study are described in Table 1.

### Human CSF samples

CSF collection methods were similar across all cohorts examined in this study. The CSF collection method from pathologically confirmed cases was as described previously in the Alzheimer’s Disease Neuroimaging Initiative procedures manual (http://www.adni-info.org/). The demographics of participants in the pathologically confirmed cohort is in Table 2. The CSF collection method from repeated LP studies was collected using the same human tau Stable Isotope Labeling Kinetics protocol as described previously^24^. The demographics of participants in the repeat LP study are described in Extended Data Table 1. The CSF collection method and demographics for the clinically diagnosed cohort were described previously^25^.

### MS analyses of MTBR-tau

Brain insoluble MTBR-tau was analyzed using filter-aided sample preparation methods as described previously^19,37^. Briefly, the whole-brain extract was incubated with 1% sarkosyl for 60 min on ice, followed by ultracentrifugation at 100,000 *g* at 4 °C for 60 min to obtain an insoluble pellet. Insoluble brain fractions were filtered, digested, desalted and injected into the mass spectrometer for analysis.

CSF MTBR-tau was analyzed as described previously^19^ with the following modifications: master mix containing detergent and chaotropic reagents (final 1% NP-40, 5 mM guanidine, protease inhibitor cocktail) and internal standards for tau (^15^N-labeled 2N4R recombinant tau) were prepared in polypropylene tubes before CSF addition. Then, 0.5 ml CSF was added and immunoprecipitated with Tau1, HJ8.5 and HJ8.7 anti-tau antibodies with epitopes residing in the N terminal to mid-domain of tau^24,25^. Post-immunoprecipitated samples depleted of the N terminal to mid-domain of tau were sequentially immunoprecipitated with 77G7 anti-tau antibody to the MTBR (residue 316–335) to measure MTBR-tau species. After washing, samples were digested with trypsin, desalted and analyzed by Orbitrap Eclipse mass spectrometer (Thermo Fisher Scientific). The MS methods used to measure MTBR-tau were as described previously with some modifications^19^. To account for individual variability in t-tau concentrations, we used the ratio of MTBR-tau_275_ or MTBR-tau_282_ normalized to the mid-domain tau peptides (181–190 and 212–221 for the brain and CSF analyses, respectively) that are common to all isoforms.

### Statistics

All statistical analyses were performed using Prism v.9.3.1 (GraphPad Software). Differences in biomarker values were assessed with a one-way analysis of variance (ANOVA) unless otherwise specified. A two-sided *P* < 0.05 was considered statistically significant and corrected for multiple comparisons using the Benjamini–Hochberg false discovery rate (FDR) method with FDR set at 5%^38^. The *P* values reported in the tables and figures were corrected using the Benjamini–Hochberg method (FDR = 5%). Spearman correlations were used to assess the associations between CSF tau biomarkers and neuropathological changes.

### Reporting summary

Further information on research design is available in the Nature Portfolio Reporting Summary linked to this article.

## Online content

Any methods, additional references, Nature Portfolio reporting summaries, source data, extended data, supplementary information, acknowledgements, peer review information; details of author contributions and competing interests; and statements of data and code availability are available at 10.1038/s41591-022-02075-9.

### Supplementary information

Supplementary Information Supplementary Figs. 1–6.

Reporting Summary

## Figures and Tables

**Extended Data Table 1:**
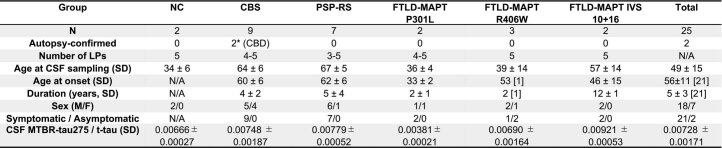
Demographic and CSF MTBR-tau measures of participants in repeated lumbar puncture cohort

NC: normal control, CBS: corticobasal syndrome, CBD: corticobasal degeneration, PSP-RS: progressive supranuclear palsy-Richardson’s syndrome, FTLD*-*MAPT: frontotemporal lobar degeneration with *MAPT* mutation, LP: lumbar puncture. *Originally enrolled as PSP-RS, later confirmed by neuropathology to be CBD.

**Extended Data Table 2:**
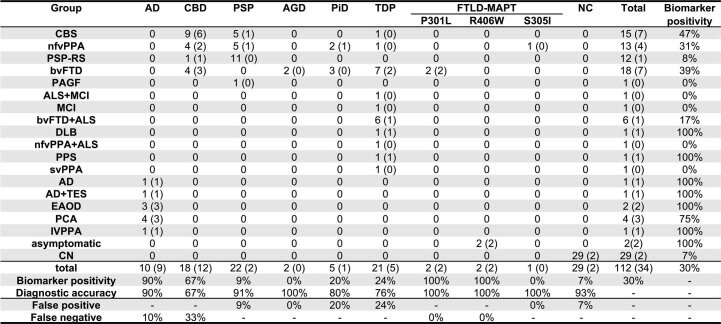
CSF MTBR-tau_275_/t-tau biomarker positivity based on clinical syndromes in pathologically-confirmed cohort

Numbers in parenthesis indicate individuals with CSF MTBR-tau_275_/t-tau decreased compared to cutoff value (0.00563). NC: normal control, FTLD-TDP: frontotemporal lobar degeneration with TAR DNA-binding protein, PiD: Pick’s disease, AGD: argyrophilic grain disease, PSP: progressive supranuclear palsy, CBD: corticobasal degeneration, FTLD*-*MAPT: frontotemporal lobar degeneration with *MAPT* mutations (P301L, S305I), bvFTD: behavioral variant of frontotemporal dementia, CBS: corticobasal syndrome, nfvPPA: nonfluent variant primary progressive aphasia, PAGF: pure akinesia with gait freezing, PSP-RS: Progressive supranuclear palsy with Richardson’s Syndrome, CN: cognitively normal.

**Extended Data Table 3:**
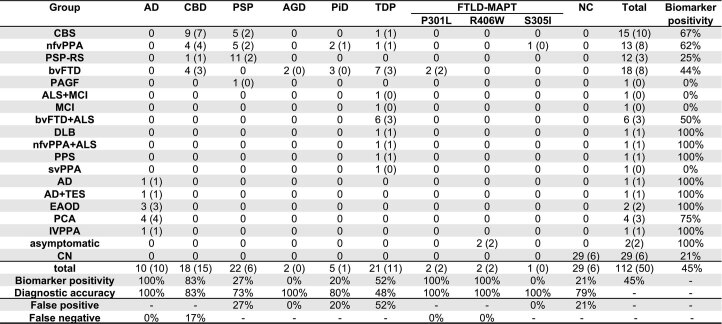
CSF MTBR-tau_282_/t-tau biomarker positivity based on clinical syndromes in pathologically-confirmed cohort

Numbers in parenthesis indicate individuals with CSF MTBR-tau_282_/t-tau decreased compared to cutoff value (0.01220). NC: normal control, FTLD-TDP: frontotemporal lobar degeneration with TAR DNA-binding protein, PiD: Pick’s disease, AGD: argyrophilic grain disease, PSP: progressive supranuclear palsy, CBD: corticobasal degeneration, FTLD*-*MAPT: frontotemporal lobar degeneration with *MAPT* mutations (P301L, S305I), bvFTD: behavioral variant of frontotemporal dementia, CBS: corticobasal syndrome, nfvPPA: nonfluent variant primary progressive aphasia, PAGF: pure akinesia with gait freezing, PSP-RS: Progressive supranuclear palsy with Richardson’s Syndrome, CN: cognitively normal.

## Data Availability

The datasets generated and/or analyzed during the current study are available from the corresponding author (C.S.). The data that support the findings of this study are not openly available due to the human data, which is stored in a controlled access repository. We will share datasets within the restrictions of institutional review board ethics approvals, upon reasonable request.
