# Supplementary material for: CSF tau microtubule-binding region identifies pathological changes in primary tauopathies
Source: Nat Med. 2022 Nov 24;28(12):2547–54. doi: 10.1038/s41591-022-02075-9 (PMC9800273; doi:10.1038/s41591-022-02075-9)
Supplement: Supplementary file 1 — Supplementary Figs. 1–6. [file 41591_2022_2075_MOESM1_ESM.pdf]

nature medicine

Article

https://doi.org/10.1038/s41591-022-02075-9

CSF tau microtubule-binding region identifies pathological changes in primary tauopathies

In the format provided by the authors and unedited

Supplementary Information

CSF tau microtubule binding region identifies pathological changes in primary tauopathies

Kanta Horie, Ph.D.[1,2], Nicolas R. Barthélemy, Ph.D. [1,2], Salvatore Spina, M.D., Ph.D. [3], Lawren VandeVrede, M.D., Ph.D.[3], Yingxin He, Ph.D.[1,2], Ross W. Paterson, M.D., Ph.D.[4], Brenton A. Wright M.D., Ph.D.,[5], Gregory S. Day, M.D., M.Sc, MSCI,[6], Albert A. Davis, M.D., Ph.D.,[1,7], Celeste M. Karch, Ph.D.[7,8,9], William W. Seeley., M.D., Ph.D. [3], Richard J. Perrin, M.D., Ph.D.[1,7,9,10], Rama K. Koppisetti, M.Sc[1,8], Faris Shaikh[1], Argentina Lario Lago, Ph.D.,[3], Hilary W. Heuer, Ph.D.,[3], Nupur Ghoshal, M.D., Ph.D.,[1,8], Audrey Gabelle, M.D., Ph.D.[11], Bruce L. Miller, M.D.[3], Adam L. Boxer, M.D., Ph.D.,[3], Randall J. Bateman, M.D.,*[1,2,7,9] , and Chihiro Sato, Ph.D.,*[1,2]

[1]Department of Neurology, Washington University School of Medicine, St. Louis, MO 63110, USA

[2]The Tracy Family SILQ Center, Washington University School of Medicine, St. Louis, MO, 63110

[3]Department of Neurology, University of California San Francisco, San Francisco, CA 94158, USA

[4] Department of Neurology, UCL Queen Square Institute of Neurology, University College London, UK

[5]University of California San Diego School of Medicine, La Jolla, CA, 92093

[6]Department of Neurology, Mayo Clinic Florida, Jacksonville, FL 32224, USA

[7]Hope Center for Neurological Disorders

[8]Department of Psychiatry, Washington University School of Medicine, St. Louis, MO 63110, USA

[9]Charles F. and Joanne Knight Alzheimer Disease Research Center, Washington University School of Medicine, St. Louis, MO 63110, USA

[10]Department of Pathology and Immunology, Washington University School of Medicine, St. Louis, MO 63110, USA

[11]Memory Research and Resources Center, Department of Neurology, University Hospital of Montpellier, Neurosciences Institute of Montpellier, University of Montpellier, France

*To whom correspondence should be addressed:

Chihiro Sato, Ph.D.,

Washington University School of Medicine

660 S Euclid Ave Campus Box 8111

St. Louis, MO 63110

Phone: 314-273-7734

Email: satochihiro@wustl.edu

Randall J Bateman, M.D.,

Washington University School of Medicine

660 S Euclid Ave Campus Box 8111

St. Louis, MO 63110

Phone: 314-747-7066

Email: batemanr@wustl.edu

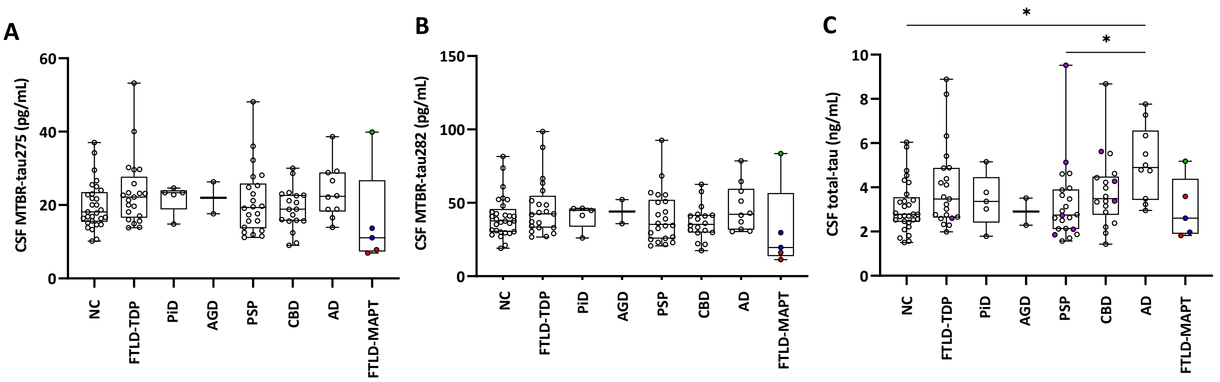

Supplementary Fig 1. CSF MTBR-tau and t-tau concentrations do not reflect CBD and FTLD-MAPT pathologies. CSF MTBR-tau275 (A) and MTBR-tau282 (B) concentrations do not change with tauopathies (n=112 total including 29 NC). FTLD-MAPT includes P301L (red, n=2), R406W (blue, n=2) and S305I (green, n=1). CSF t-tau (C) increased in autopsy confirmed AD (n=10) compared to NC (n=29) and PSP (n=22, $p<0.05$). Significance in statistical test: *$P<0.05$. The box plots show the minimum, 25 percentile, median, 75 percentile, and maximum. Differences in biomarker values were assessed with one-way ANOVAs. A two-sided $p<0.05$ was considered statistically significant and corrected for multiple comparisons using Benjamini-Hochberg false discovery rate (FDR) method with FDR set at 5%. NC: normal control, FTLD-TDP: frontotemporal lobar degeneration with TAR DNA-binding protein, PiD: Pick's disease, AGD: argyrophilic grain disease, PSP: progressive supranuclear palsy, CBD: corticobasal degeneration, AD: Alzheimer's disease, FTLD-MAPT: frontotemporal lobar degeneration with *MAPT* mutation.

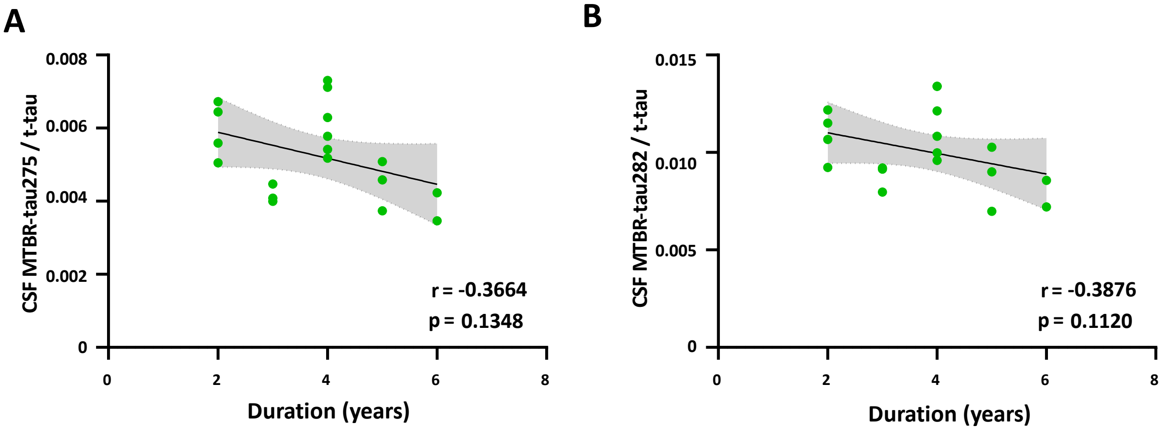

Supplementary Fig. 2. CSF MTBR-tau/tau correlates with disease duration in CBD. CSF MTBR-tau275/t-tau (A) and MTBR-tau282/t-tau (B) negatively correlates with duration of the disease in CBD (i.e. interval between age of onset and CSF collection, n=18, Spearman r=-0.37, p=0.13 and r=-0.39, p=0.11, respectively). Gray shadow represents 95% confidential intervals for linear regression. CBD: corticobasal degeneration.

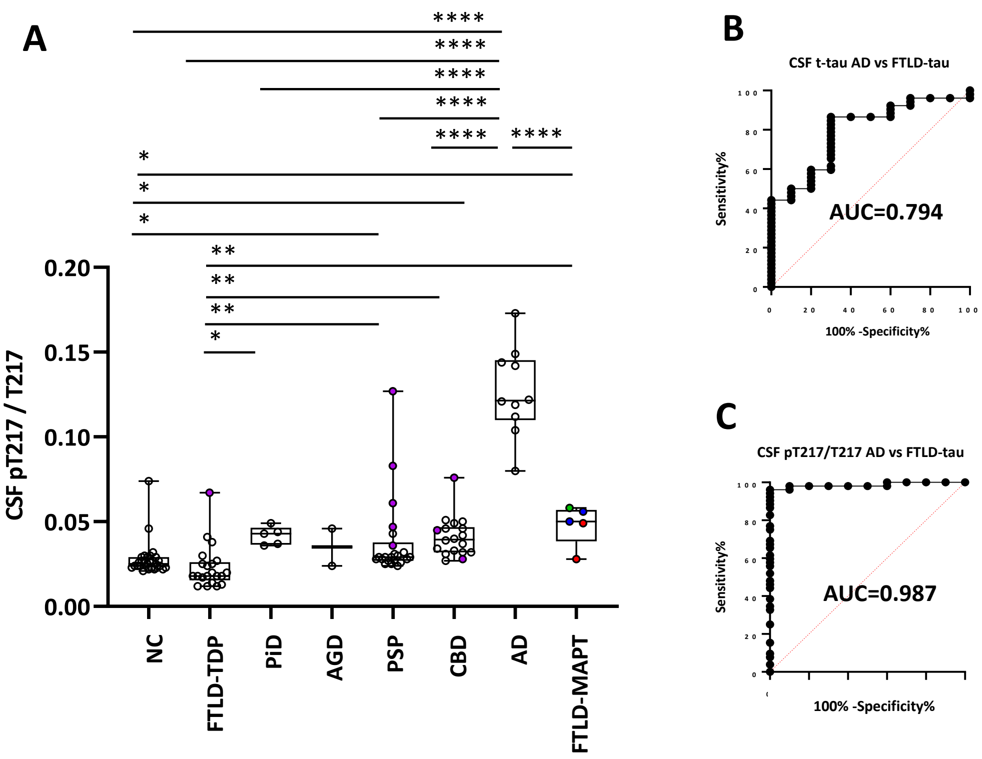

Supplementary Fig. 3. CSF pT217/T217 identifies AD from other tauopathies. (A) CSF pT217/T217 increased in AD (n=10) compared to NC (n=29), FTLD-tau (i.e. PiD (n=5), PSP (n=22), CBD (n=18), and FTLD-MAPT (n=5)), and FTLD-TDP (n=21) (p<0.0001). PSP, CBD, and FTLD-TDP with AD co-pathology (filled purple, n=9) had higher CSFpT217/T217. FTLD-MAPT includes P301L (red, n=2), R406W (blue, n=2) and S305I (green, n=1). CSF pT217/T217 decreased in FTLD-TDP (n=21) compared to PiD (n=5), PSP (n=22), CBD (n=18) and FTLD-MAPT (n=5, p<0.05-0.01). CSF t-tau (C) and CSF pT217/T217 (D) can distinguish AD (n=10) from FTLD-MAPT (n=5) with AUC=0.794, 0.987, respectively. Differences in biomarker values were assessed with one-way ANOVAs. A two-sided p<0.05 was considered statistically significant and corrected for multiple comparisons using Benjamini-Hochberg false discovery rate (FDR) method with FDR set at 5%. Significance in statistical test: ****P<0.0001, **P<0.01, *P<0.05. NC: normal control, FTLD-TDP: frontotemporal lobar degeneration with TAR DNA-binding protein, PiD: Pick's disease, AGD: argyrophilic grain disease, PSP: progressive

supranuclear palsy, CBD: corticobasal degeneration, AD: Alzheimer’s disease, FTLD-MAPT: frontotemporal lobar degeneration with *MAPT* mutation. AUC: area under the curve.

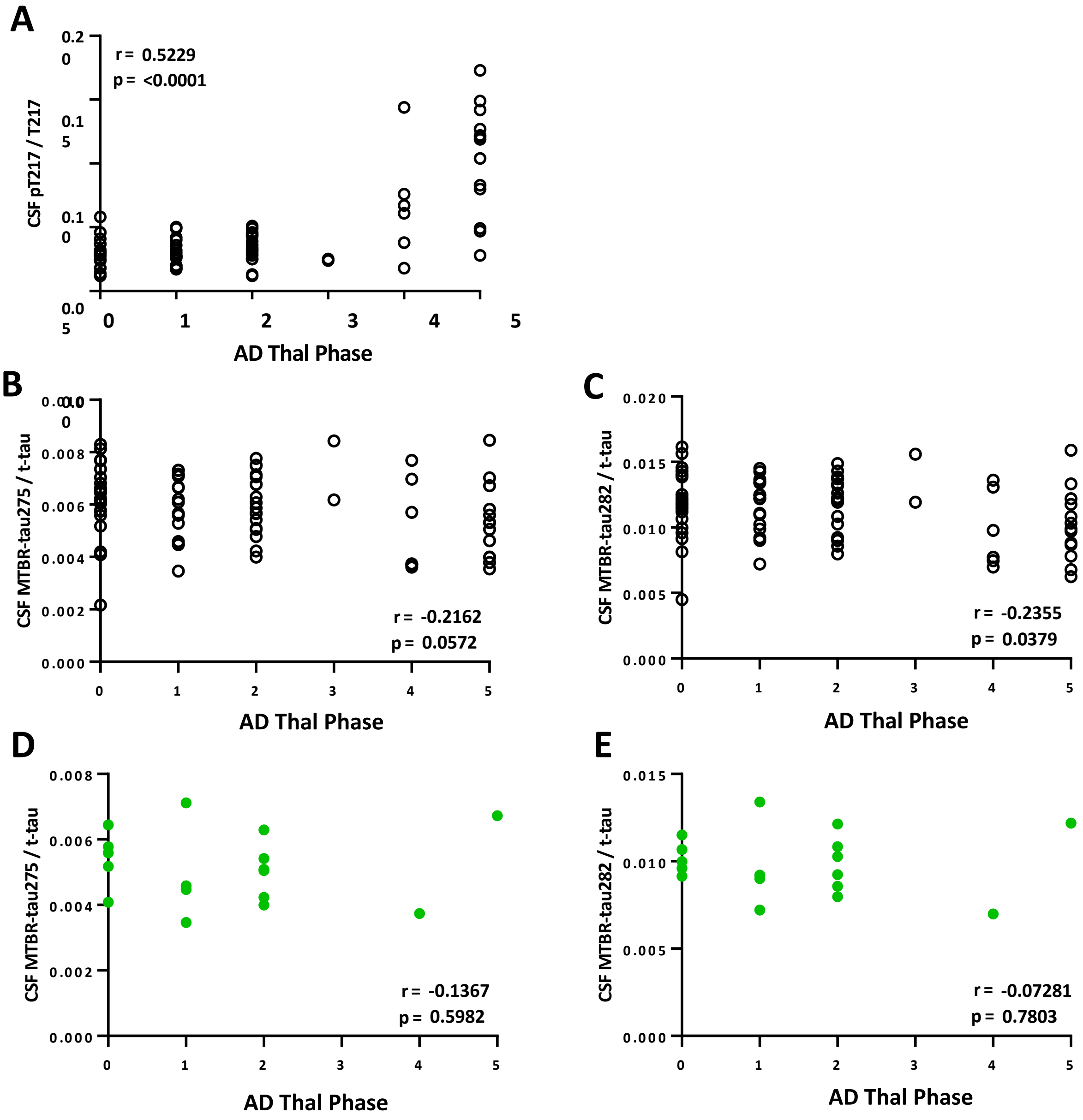

Supplementary Fig. 4. CSF MTBR-tau275/t-tau and MTBR-tau282/t-tau do not correlate with amyloid pathology in primary tauopathies. (A) CSF pT217/T217 positively correlates with AD Thal Phase (Spearman r=0.52, p<0.0001). CSF MTBR-tau275/t-tau (B, D) and CSF MTBR-tau282/t-tau (C, E) do not correlate with AD Thal Phase measured in autopsied brains of all tauopathy cohort (B, C, n=79) or CBD only (D, E, n=17).

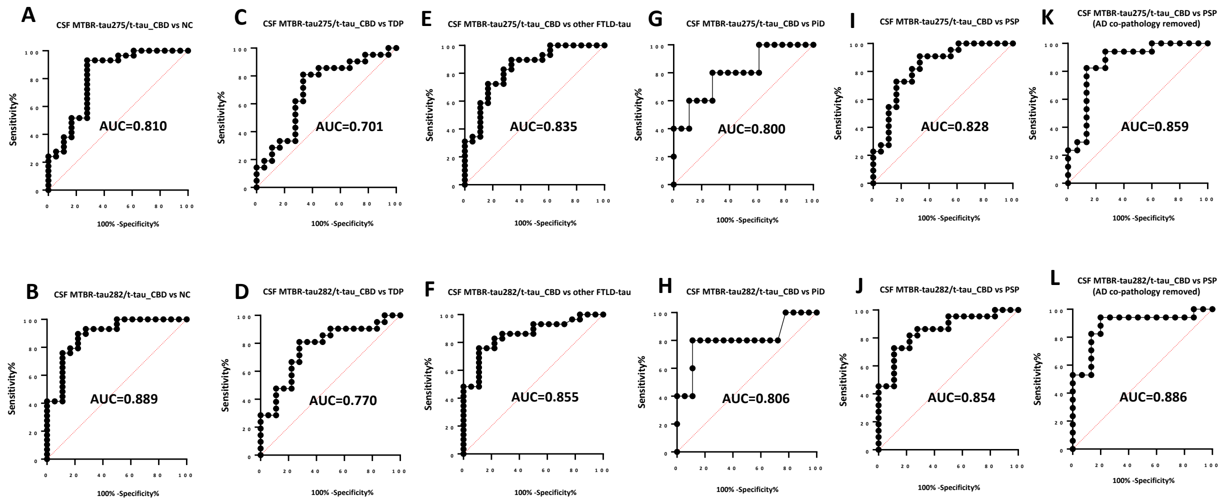

Supplementary Fig. 5. ROC curves for 4R specific CSF MTBR-tau to distinguish CBD from control and other tauopathies. CSF MTBR-tau275/t-tau (A, C, E, G, I, K) and MTBR-tau282/t-tau (B, D, F, H, J, L) can distinguish CBD from NC (A, B), FTLD-TDP (C, D), FTLD-tau (i.e. PSP, PiD and AGD, E, F), PiD (G, H), and PSP (I, J). AUC improves when AD co-pathology cases are excluded from CBD and PSP (K, L). NC: normal control, FTLD-TDP: frontotemporal lobar degeneration with TAR DNA-binding protein, PiD: Pick's disease, AGD: argyrophilic grain disease, PSP: progressive supranuclear palsy, CBD: corticobasal degeneration, AD: Alzheimer's disease, FTLD-MAPT: frontotemporal lobar degeneration with *MAPT* mutation. AUC: area under the curve, ROC: receiver operating characteristic.

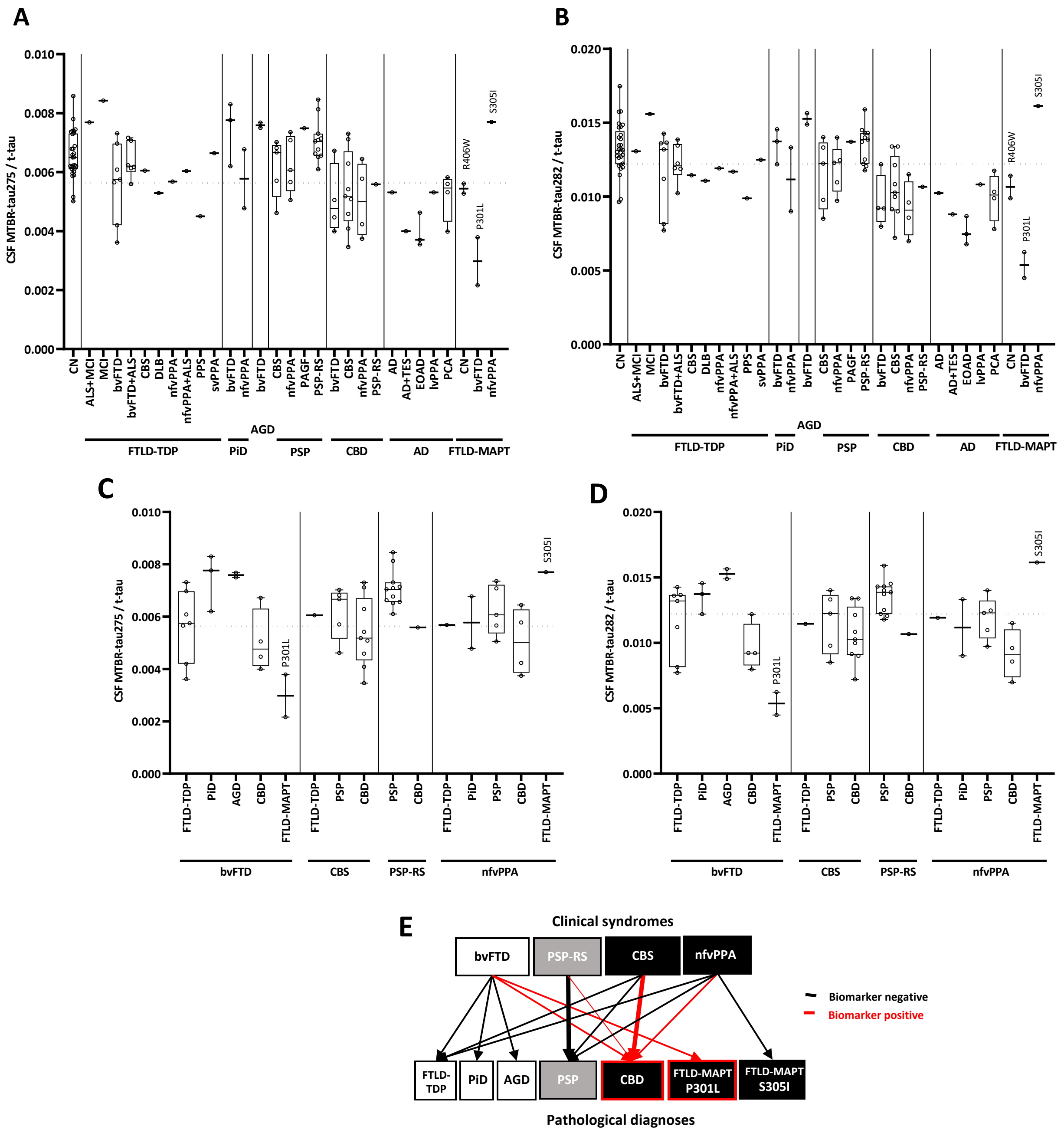

Supplementary Fig. 6. Retrospective clinical syndromes and CSF MTBR-tau markers in pathologically-confirmed cohort. CSF MTBR-tau275/t-tau (A, C) and MTBR-tau282/t-tau (B, D) by pathological diagnoses (A, B) and clinical syndromes (C, D) are shown (total n=112). The box plots show the minimum, 25 percentile, median, 75 percentile, and maximum. Dotted lines show cutoff of 0.00563 and 0.01220, respectively for CSF MTBR-tau275/t-tau and MTBR-

tau282/t-tau. (E) Schematic of relationship between clinical syndrome and pathological diagnoses in FTLD. Red and black lines show CSF MTBR-tau biomarkers positive and negative, respectively. Biomarker positivity was determined by the median of each disease group. NC: normal control, FTLD-TDP: frontotemporal lobar degeneration with TAR DNA-binding protein, PiD: Pick's disease, AGD: argyrophilic grain disease, PSP: progressive supranuclear palsy, CBD: corticobasal degeneration, FTLD-MAPT: frontotemporal lobar degeneration with *MAPT* mutations (P301L, S305I), bvFTD: behavioral variant of frontotemporal dementia, CBS: corticobasal syndrome, nfvPPA: nonfluent variant primary progressive aphasia, PAGF: pure akinesia with gait freezing, PSP-RS: Progressive supranuclear palsy with Richardson's Syndrome, CN: cognitively normal.
