# Supplementary material for: CSF tau microtubule-binding region identifies pathological changes in primary tauopathies
Source: Nat Med. 2022 Nov 24;28(12):2547–54. doi: 10.1038/s41591-022-02075-9 (PMC9800273; doi:10.1038/s41591-022-02075-9)
Supplement: Supplementary file 2 — Reporting Summary [file 41591_2022_2075_MOESM2_ESM.pdf]

nature portfolio

Corresponding author(s): Chihiro Sato & Randall Bateman

Last updated by author(s): 2022-09-27

Reporting Summary

Nature Portfolio wishes to improve the reproducibility of the work that we publish. This form provides structure for consistency and transparency in reporting. For further information on Nature Portfolio policies, see our Editorial Policies and the Editorial Policy Checklist.

Statistics

For all statistical analyses, confirm that the following items are present in the figure legend, table legend, main text, or Methods section.

n/a	Confirmed	
☐	☒	The exact sample size (*n*) for each experimental group/condition, given as a discrete number and unit of measurement
☐	☒	A statement on whether measurements were taken from distinct samples or whether the same sample was measured repeatedly
☐	☒	The statistical test(s) used AND whether they are one- or two-sided<br>*Only common tests should be described solely by name; describe more complex techniques in the Methods section.*
☒	☐	A description of all covariates tested
☒	☐	A description of any assumptions or corrections, such as tests of normality and adjustment for multiple comparisons
☐	☐	A full description of the statistical parameters including central tendency (e.g. means) or other basic estimates (e.g. regression coefficient) AND variation (e.g. standard deviation) or associated estimates of uncertainty (e.g. confidence intervals)
☐	☒	For null hypothesis testing, the test statistic (e.g. *F*, *t*, *r*) with confidence intervals, effect sizes, degrees of freedom and *P* value noted<br>*Give P values as exact values whenever suitable.*
☒	☐	For Bayesian analysis, information on the choice of priors and Markov chain Monte Carlo settings
☒	☐	For hierarchical and complex designs, identification of the appropriate level for tests and full reporting of outcomes
☐	☒	Estimates of effect sizes (e.g. Cohen's *d*, Pearson's *r*), indicating how they were calculated

Our web collection on statistics for biologists contains articles on many of the points above.

Software and code

Policy information about availability of computer code

Data collection	NanoLC-MS/MS experiments wer performed using nanoAcquity UPLC system (Waters, Mildford, Massachusetts) coupled to Orbitrap Eclipse mass spectrometer (Thermo Scientific, San Jose, California).
Data analysis	All statistical analyses were performed using GraphPad Prism software (v9.3.1). MS transitions were extracted using Skyline software (MacCoss lab, University of Washington).

For manuscripts utilizing custom algorithms or software that are central to the research but not yet described in published literature, software must be made available to editors and reviewers. We strongly encourage code deposition in a community repository (e.g. GitHub). See the Nature Portfolio guidelines for submitting code & software for further information.

Data

Policy information about availability of data

All manuscripts must include a data availability statement. This statement should provide the following information, where applicable:

- Accession codes, unique identifiers, or web links for publicly available datasets
- A description of any restrictions on data availability
- For clinical datasets or third party data, please ensure that the statement adheres to our policy

The datasets generated and/or analyzed during the current study are available from the corresponding author (CS). The data that support the findings of this study are not openly available due to the human data, which is stored in a controlled access repository. We will share datasets within the restrictions of IRB ethics approvals, upon reasonable request.

Human research participants

Policy information about studies involving human research participants and Sex and Gender in Research.

Ethics oversight	For the retrospective study of pathologically-confirmed cohort, collection and use of biospecimens was approved by the institutional review board at ALLFTD or each research center from which the individual was recruited, and this study was approved by the Biospecimen Resource Committee at UCSF. Repeated lumbar puncture (LP) study (NCT03545126) was approved by the Institutional Review Board at Washington University in St. Louis, MO, USA and University College London (UCL), UK. The study of clinically diagnosed cohort was approved by the Ethics Committee of the Montpellier University Hospital, France (CSF-Neurobank #DC-2008-417 at the certified NFS 96-900 CHU resource center BB-0033-00031 (http://www.biobanques.eu). Authorization to handle personal data was granted by the French Data Protection Authority (CNIL) under the number 1709743 v0.
Population characteristics	Participants (average age 49 ± 15) with clinical diagnoses of CBS and PSP-RS, and families with or without known MAPT mutations (P301L, R406W, and IVS10+16) were included in the repeat LP study. This study required commitment to 4-6 visits within approximately 4 months, therefore, the study population was mostly representative of the background population in the healthcare region from which the participants were recruited and reflective of known prevalence of MAPT mutations among White families.
Recruitment	Participants with clinical diagnoses of CBS and PSP-RS, asymptomatic and symptomatic individuals from known MAPT mutation families were referred locally, nationally, and internationally to be enrolled in the repeated LP study at Washington University and at UCL. Exclusion criteria included any contraindications for LPs or lumbar catheters, including a bleeding disorder, active anticoagulation, and active infection, which is not likely to impact results on CSF tau biomarkers.
Reporting of sex and gender	Both sexes were enrolled in the study and sex is self-reported during recruitment.

Note that full information on the approval of the study protocol must also be provided in the manuscript.

Field-specific reporting

Please select the one below that is the best fit for your research. If you are not sure, read the appropriate sections before making your selection.

☒ Life sciences ☐ Behavioural & social sciences ☐ Ecological, evolutionary & environmental sciences

For a reference copy of the document with all sections, see nature.com/documents/nr-reporting-summary-flat.pdf

Life sciences study design

All studies must disclose on these points even when the disclosure is negative.

Sample size	Based on preliminary result, we had effect sizes varying from 0.48-0.49 for the comparisons among primary tauopathy, AD and control, and 0.059-0.43 for comparisons between CBD, FTLD and PSP (one-way ANOVA). The two highest effect sizes are (A) 0.34 and (B) 0.43 for the three subgroups in primary tauopathies. If we had CBD:FTLD:PSP = 20:20:20, we were (A) 60% or (B) 80% powered to detect differences between CBD:FTLD:PSP, and (A) 98% and (B) 93% powered to detect differences between primary tauopathies vs AD vs Control. We obtained 22 PSP, 18 CBD, and 21 FTLD-TDP that were available
Data exclusions	We excluded one participant data from "pathologically confirmed cohort" because the participant did not have the pathological diagnosis while the cognitive impairment was observed (MMSE=22).
Replication	All CSF samples from the current study were analyzed together with QC controls CSF to monitor inter-assay variability for each variable at low, medium and high CSF tau and ptau, which confirmed high reproducibility. Due to the nonrenewable status of the CSF from each cohort study, only the absolute minimum was requested and approved by each institute, precluding the reanalyses of these samples with mass spectrometry experiments.
Randomization	Samples were randomized by genetic status (mutation carrier or non-carrier), neuropathological results (i.e. CBD, PSP, FTLD) or clinical syndromes (i.e. CBS, PSP-RS, FTD). All samples had a random code as an identifier and researchers who performed experiments were blinded towards the code when performing brain or CSF analyses.
Blinding	The researchers conducting brain and CSF analyses were blinded to neuropathological and clinical data and group assignment.

Reporting for specific materials, systems and methods

We require information from authors about some types of materials, experimental systems and methods used in many studies. Here, indicate whether each material, system or method listed is relevant to your study. If you are not sure if a list item applies to your research, read the appropriate section before selecting a response.

Materials & experimental systems

n/a	Involved in the study
☐	☒ Antibodies
☒	☐ Eukaryotic cell lines
☒	☐ Palaeontology and archaeology
☒	☐ Animals and other organisms
☐	☒ Clinical data
☒	☐ Dual use research of concern

Methods

n/a	Involved in the study
☒	☐ ChIP-seq
☒	☐ Flow cytometry
☒	☐ MRI-based neuroimaging

Antibodies

Antibodies used	Tau1 (generated by Dr. Nicholas Kanaan), HJ8.5, HJ8.7 (generated by Dr. David Holtzman), and 77G7 (Biolegend 816703) tau antibodies. 3mg antibody/gram Sepharose beads were generated and 11.25ug antibody/sample was used for immunoprecipitation.
Validation	Tau1 antibody (AB_2721197) was validated in Binder et al., 1985, and Sato et al., 2018. HJ8.5 (AB_2721237) and HJ8.7 (AB_2721234) antibodies were validated in Yanamandra et al., 2013. 77G7 antibody (AB_2728535) is described in: https://www.biolegend.com/en-us/products/purified-anti-tau-316-355-antibody-11567. The assay of each tau species using the immunoprecipitation followed by mass spectrometry analysis has been validated in previously studies, including Sato et al., 2018, Barthelemy et al., 2019; Horie et al., 2021.

Clinical data

Policy information about clinical studies
All manuscripts should comply with the ICMJE guidelines for publication of clinical research and a completed CONSORT checklist must be included with all submissions.

Clinical trial registration	NCT03545126.
Study protocol	Repeated LP study is an observational study. https://clinicaltrials.gov/ct2/show/NCT03545126?term=Randall+Bateman&draw=2&rank=3
Data collection	Data was obtained from CSF from repeated lumbar punctures in 25 participants with CBS, PSP-RS, or with or without FTLD-MAPT mutations.
Outcomes	Secondary outcome was used in this study that included concentration of CSF tau, measured using tau protein isoforms that are immunoprecipitated and analyzed by mass spectrometry.
